# Environment-dependent chlorophyll–chlorophyll charge transfer states in Lhca4 pigment–protein complex

**DOI:** 10.3389/fpls.2024.1412750

**Published:** 2024-08-07

**Authors:** Gabrielė Rankelytė, Andrius Gelzinis, Bruno Robert, Leonas Valkunas, Jevgenij Chmeliov

**Affiliations:** ^1^ Institute of Chemical Physics, Faculty of Physics, Vilnius University, Vilnius, Lithuania; ^2^ Department of Molecular Compound Physics, Center for Physical Sciences and Technology, Vilnius, Lithuania; ^3^ Université Paris-Saclay, CEA, CNRS, Institute for Integrative Biology of the Cell, Gif-sur-Yvette, France

**Keywords:** LHCI, chlorophyll, amino acid, atomic partial charge, quantum chemistry, charge transfer states

## Abstract

Photosystem I (PSI) light-harvesting antenna complexes LHCI contain spectral forms that absorb and emit photons of lower energy than that of its primary electron donor, P700. The most red-shifted fluorescence is associated with the Lhca4 complex. It has been suggested that this red emission is related to the inter-chlorophyll charge transfer (CT) states. In this work we present a systematic quantum-chemical study of the CT states in Lhca4, accounting for the influence of the protein environment by estimating the electrostatic interactions. We show that significant energy shifts result from these interactions and propose that the emission of the Lhca4 complex is related not only to the previously proposed *a*603^+^–*a*608^−^ state, but also to the *a*602^+^–*a*603^−^ state. We also investigate how different protonation patterns of protein amino acids affect the energetics of the CT states.

## 1 Introduction

In oxygenic photosynthesis, the storage of the light energy in the form of chemical bonds requires two photosystems: photosystem II (PSII), which extracts electrons from water, and photosystem I (PSI), which produces the low redox potential electrons that ultimately reduce NADP into NADPH. In all organisms, PSI is constituted of a huge multi-protein architecture that binds a large number of chlorophylls and ensures a photo-induced electron transfer reactions across the photosynthetic membrane. In higher plants and green algae, the core of PSI is associated with an ensemble of light-harvesting chlorophyll–protein complexes, termed LHCI antennae (Haworth et al., 1983). In the early 80’s, it was shown that PSI in *Chlamydomonas* binds two types of LHCI proteins: one fluorescing at 686 nm and the other around 715 nm (Ish-Shalom and Ohad, 1983). Later, a similar situation was observed in higher plants as well, with even longer-wavelength (around 730 nm) emission of the redder spectral species (Lam et al., 1984). These two observed complexes with distinct fluorescence spectra are actually composed of four polypeptides, Lhca1–4, belonging to the LHC superfamily, which all bind chlorophylls *a* and *b*. Electron microscopy studies showed that LHCI complexes bind on one side of the PSI core (Boekema et al., 2001), and later X-ray crystallographic studies allowed a precise description of the LHCI–PSI core interactions (Ben-Shem et al., 2003).

Initially, the heterodimer comprised of the Lhca1 and Lhca4 complexes was shown to be responsible for the observed red emission (Knoetzel et al., 1992). More recent work, however, actually revealed that the native heterodimer formed by other two complexes, Lhca2/3, also displays a red-shifted emission (Wientjes and Croce, 2011). Because of distinct spectroscopic differences from the major light-harvesting complexes of PSII, LHCII, the LHCI antennae in general and its constituents Lhca1 and Lhca4 in particular attracted intense attention. Particularly, it was noted that the excited state energies of some of LHCI Chls are lower than that of the PSI primary electron donor, which, albeit extending the PSI absorption toward the red spectral region, also slows down the excited energy trapping by the reaction center. The mechanisms underlying this spectral red shift had to be clarified.

The study of these red forms largely benefited from approaches using LHC reconstitution (Plumley and Schmidt, 1987). Reconstituted complexes indeed led to the conclusion that the Chl molecules displaying highly red-shifted fluorescence exclusively belong to the Lhca4 complex (Croce et al., 2002). Moreover, reconstitution of the pertinently mutated Lhca4 showed that the red fluorescence of these complexes disappears when the asparagine ligand of Chl *a*603 (using the Qin et al. (2015) notation) is replaced by the histidine (Morosinotto et al., 2003). This was taken as a straightforward indication that this Chl *a*603 (together with the neighboring Chl *b*609 it interacts with) was responsible for the nearly 50-nm fluorescence redshift. Recently, genetic engineering of Lhca4 in *Arabidopsis* fully supported the results obtained from the reconstituted complexes (Li et al., 2023).

Detailed spectroscopic characterization of Lhca4 reconstituted from wild type and mutated polypeptides showed that the low-energy electronic transitions display a large bandwidth, which were proposed to arise from the mixing of an excitonic state, involving Chl *a*603 and Chl *b*609 molecules, with an inter-pigment charge transfer (CT) state (Croce et al., 2007). The determination of the PSI structure from higher plants at high resolution (Ben-Shem et al., 2003; Mazor et al., 2015; Qin et al., 2015, 2017) opened the way to model the electronic structure of LHCI complexes and Lhca4 in particular. By performing molecular dynamics simulations using the Quantum mechanics/Molecular mechanics (QM/MM) approach, the influence of a possible CT state in the Chl *a*603/*b*609 exciton manifold was investigated (Sláma et al., 2023). It was showed that such a state is highly sensitive to the precise relative geometry of the Chl pair, also explaining its disappearance in the asparagine mutant. Using an exciton model including a CT state, Novoderezhkin and Croce (2023) were able to simulate the ensemble of Lhca4 electronic properties on the basis of an exciton-type mixing of a CT state with the excited state manifold.

In this paper, we also aim to characterize the CT states present in Lhca4 complex using quantum mechanical calculations. The CT states are considered only energetically, and possible mixing with the excitonic states of the pigments is not included. However, we do not focus solely on the Chl *a*603/*b*609 pair but rather try to pinpoint the ensemble of possible CT states present in this complex. Indeed, the asparagine-to-histidine (N98H, based on the notation of Qin et al. (2015)) mutation is likely to eventually perturb not only this pair, and, as underlined by Sláma et al. (2023), even minimal structural changes may result in dramatic changes in CT states manifold. Moreover, a second red form of Lhca4 exhibiting emission at ~705 nm exists, which is also present in the N98H mutant. Although the atomic structure of this complex is known, there are still some parameter values that should be specified in order to obtain a unique model of this system. Among these parameters, the exact protonation state of the protonable aminoacids in Lhca4 remains relatively poorly investigated. Accordingly, we tested the influence of several possible protonation states on the energetics of the CT states in this complex. Our results show that the *a*602^+^–*a*603^−^ state should also be related to the far-red fluorescence. Moreover, (de)protonation of a few amino acids could significantly alter the CT state energetics.

## 2 Methods

### 2.1 Quantum chemical calculations

**Figure 1:**
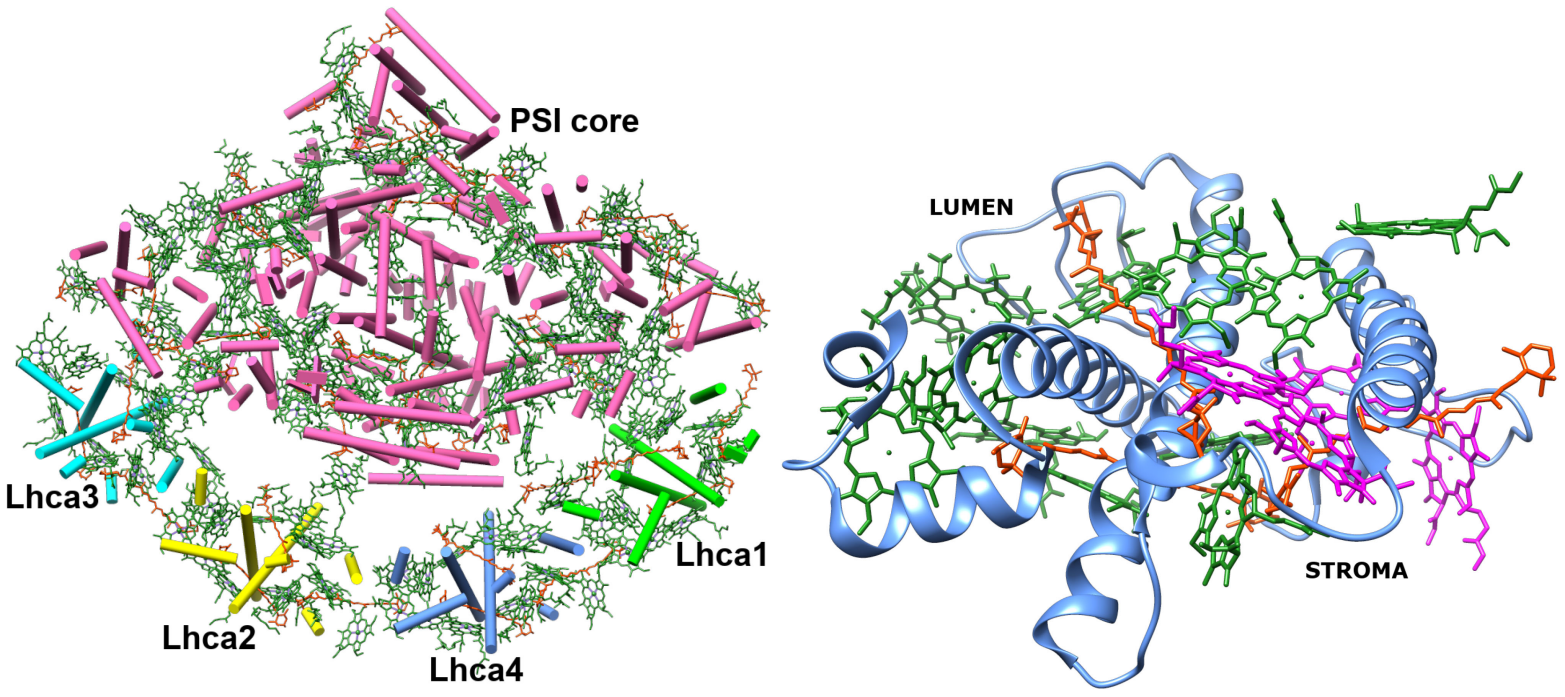
Left: PSI–LHCI supercomplex (PDB ID: 4XK8) (Qin et al., 2015). Right: Lhca4 light-harvesting complex. Chlorophylls *a* are depicted in green and Chlorophylls *b* are depicted in magenta. Carotenoids (BCR, XAT and LUT) are depicted in orange.

The structure of Lhca4 was obtained as the 4th chain of the pea PSI–LHCI supercomplex structure (Qin et al., 2015) (PDB ID: 4XK8) from the PDB database (see **Figure 1**). In this work, we seek to investigate the energetics of the inter-chlorophyll charge transfer states that are expected to be present in this complex. Short distance between the pigments in the pigment–protein complex is a prerequisite for a CT state formation. In order to find possible locations of the CT states in Lhca4, we first determined which chlorophyll dimers have the shortest (up to 12 Å) Mg–Mg distance and found 12 dimers that satisfy this condition.

For all single chlorophylls that form these dimers, geometry optimization *in vacuo* was performed. The phytyl tail of these chlorophylls was truncated by replacing the phytyl group with the methyl group, similarly to the previous works (Jornet-Somoza et al., 2015; Sirohiwal et al., 2020a; Mikalčiūtė et al., 2022), in order to reduce the calculation time and prevent extreme structural changes of the tail during the geometry optimization. Missing hydrogen atoms were added using GaussView software (Dennington et al., 2019). Ground state geometry optimization was performed using Gaussian 16 (Frisch et al., 2016). Density Functional Theory (DFT) calculations were performed using CAM-B3LYP functional (Yanai et al., 2004) and 6–31G(d) basis set. The DFT level of theory with the aforementioned functional has been commonly used for chlorophyll systems (Yin et al., 2007; Higashi et al., 2014; Frankcombe, 2015; Bold et al., 2020).

We formed 12 selected dimers by mapping individual chlorophylls with optimized geometry onto the Lhca4 structure using UCSF Chimera software (Pettersen et al., 2004). TD-DFT calculations were conducted for the lowest eight excited states of these dimers, treating them as a single supermolecule, employing the CAM-B3LYP functional and a 6–31G(d) basis set. Our examination included various parameters, such as energy, static dipole moment, transition dipole moment, and the sum of the Mulliken partial charges for each molecule in the dimer. These findings unveiled the presence of 19 potential inter-chlorophyll charge transfer states within the Lhca4 pigment–protein complex.

### 2.2 Modelling of pigment–protein complex environment

In order to account for the environment that surrounds the dimers of interest and calculate its impact on energy values of the CT states, we chose to follow the methodology developed by Renger and co-workers, which was previously applied for local excitations (Adolphs et al., 2007; Renger and Müh, 2013). The environment (protein, other single chlorophyll molecules, and carotenoids) is divided into the environmental blocks. These blocks are coupled through the Coulomb interactions. The dimer of interest m is described by the wavefunction $|A_{a}^{\left(m\right)}\rangle$ , where *a* indicates the electronic state of the dimer. The dimer is surrounded by N− 1 environmental blocks with the wavefunctions $|B_{b}^{\left(\eta \right)}\rangle$ , where η= 1 ,…, N−1 is the number of the block and b is the electronic state of this block. These wavefunctions satisfy the following Schrödinger equations:


(1) $$\begin{array}{l} H_{A}^{\left(m\right)}|A_{a}^{\left(m\right)}\rangle =E_{a}^{\left(m\right)}|A_{a}^{\left(m\right)}\rangle ,\\ H_{B}^{\left(\eta \right)}|B_{b}^{\left(\eta \right)}\rangle =F_{b}^{\left(\eta \right)}|B_{b}^{\left(\eta \right)}\rangle . \end{array}$$

Here in Equation 1 $H_{A}^{\left(m\right)}$ and $H_{B}^{\left(\eta \right)}$ are the Hamiltonians of the environmental blocks of the system *in vacuo*, $E_{a}^{\left(m\right)}$ is the energy of the *a*th electronic state of the dimer, $F_{b}^{\left(\eta \right)}$ —the energy of the *b*th electronic state of the environmental block η . The total Hamiltonian of the system is given by


(2) $$H=H_{A}^{\left(m\right)}+\sum_{\eta }H_{B}^{\left(\eta \right)}+\sum_{\eta }V_{AB}^{\left(m,\eta \right)}+\frac{1}{2}\sum_{\eta \neq \eta }V_{BB}^{\left(\eta ,\eta '\right)}.$$

Here $V_{AB}^{\left(m,\eta \right)}$ describes the Coulomb coupling between the dimer and η block and $V_{BB}^{\left(\eta ,\eta^{\prime }\right)}$ describes the interaction between two environmental blocks η and $\eta^{\prime }$ . The last two terms define the interaction Hamiltonian *V*. We assume that the blocks do not undergo electron exchange, thus the wavefunctions of the system can be combined using the Hartree product:


(3) $$|\psi_{a\text{b}}^{\left(m\right)}\rangle =|A_{a}^{\left(m\right)}\rangle \prod_{\eta }|B_{b_{\eta }}^{\left(\eta \right)}\rangle ,$$

where $|\psi_{ab}^{\left(m\right)}\rangle$ is the eigenfunction of the Schrödinger equation


(4) $$\left(H_{A}^{\left(m\right)}+\sum_{\eta }H_{B}^{\left(\eta \right)}\right)|\psi_{ab}^{\left(m\right)}\rangle =\left(E_{a}^{\left(m\right)}+F_{b}\right)|\psi_{ab}^{\left(m\right)}\rangle ,$$

$\text{b}=\left\{b_{1},\;b_{2},\;\ldots ,b_{\eta },\ldots \right\}$ enumerates the electronic states of the environment, and $F_{\text{b}}=\sum_{\eta }F_{b_{\eta }}^{\left(\eta \right)}$ . Equations 3 and 4 can be used to determine the energy shift $\Delta E_{a}^{\left(m\right)}$ that the *a*th state of the dimer acquires after experiencing the electrostatic interaction with the environmental blocks, given by the two last terms in Equation 2. Within the first order perturbation theory (the charge density coupling (CDC) method), the energy shift is given as:


(5) $$\Delta E_{a}^{\left(m\right)}=\langle \psi_{a0}^{\left(m\right)}|V|\psi_{a0}^{\left(m\right)}\rangle ,$$

here index $0=\;\{0,0{,}_{...}\}$ denotes the electronic ground state of all the environmental blocks.

The energy of the transition between the ground and the *s*th excited state is given by:


(6) $$E_{s}^{\left(m\right)}=E_{0}^{\left(m\right)}+\Delta E_{s}^{\left(m\right)}-\Delta E_{0}^{\left(m\right)}.$$

Here $E_{0}^{\left(m\right)}$ is the transition energy between the ground and the *s*th excited state *in vacuo*, $\Delta E_{s}^{\left(m\right)}$ and $\Delta E_{0}^{\left(m\right)}$ are the energy shifts for the excited and the ground state, respectively. Energy shifts of electronic state *a* of dimer *m* are determined by calculating the values of the Coulomb matrix elements given in Equation 5:


(7) $$\Delta E_{a}^{\left(m\right)}=\sum_{\eta }\langle A_{a}^{\left(m\right)}B_{0}^{\left(\eta \right)}|\;V_{AB}^{\left(m,\eta \right)}|A_{a}^{\left(m\right)}B_{0}^{\left(\eta \right)}\rangle +\frac{1}{2}\sum_{\eta \neq \eta^{\prime }}\langle B_{0}^{\left(\eta^{\prime }\right)}B_{0}^{\left(\eta \right)}|\;V_{BB}^{\left(\eta ,\eta^{\prime }\right)}|B_{0}^{\left(\eta^{\prime }\right)}B_{0}^{\left(\eta \right)}\rangle .$$

Here, in Equation 7, the first term on the right hand side denotes the Coulomb interaction between the charge densities of the dimer *m* of state *a* and the environmental block η in its ground state, and the second one denotes the Coulomb interaction between the charge densities of environmental blocks η and $\eta^{\prime }$ in the ground state. The elements of the Coulomb matrix can be approximated as the interaction between the atomic partial charges (Adolphs et al., 2007; Renger and Müh, 2013):


(8) $$\langle A_{a}^{\left(m\right)}B_{0}^{\left(\eta \right)}|V_{AB}^{\left(m,\eta \right)}|A_{a}^{\left(m\right)}B_{0}^{\left(\eta \right)}\rangle =\frac{1}{4\pi \varepsilon_{0}}\sum_{I,J}\frac{q_{I}^{\left(m\right)}(a,a)q_{J}^{(\eta )J}(0,0)}{\left|{\text{R}}_{I}^{\left(m\right)}-{\text{R}}_{J}^{\left(\eta \right)}\right|},$$

where in Equation 8 $\varepsilon_{0}$ denotes the permittivity of vacuum, $q_{I}^{\left(m\right)}(a,a)$ and $q_{J}^{\left(\eta \right)}(0,0)$ are the atomic partial charges of the *I*-th atom of the dimer and *J*-th atom of the environment, respectively. The positions of these charges are ${\text{R}}_{I}^{\left(m\right)}$ and ${\text{R}}_{J}^{\left(\eta \right)}$ .

The possible errors in the values of the atomic partial charges can be compensated by introducing the effective dielectric constant $\varepsilon_{\text{eff}}$ to scale the Coulomb interaction. Its adjustment can thus approximately account for the higher order terms not included in the CDC approximation. Then, following Equation 6, the energy of the transition between the ground and the *s*-th excited state of the dimer *m* is given as:


(9) $$\begin{array}{l} E_{s}^{\left(m\right)}=E_{0}^{\left(m\right)}+\frac{1}{\varepsilon_{\text{eff}}}\sum_{\eta }\left(\langle A_{s}^{\left(m\right)}B_{0}^{\left(\eta \right)}|V_{AB}^{\left(\eta \right)}|A_{s}^{\left(m\right)}B_{0}^{\left(\eta \right)}\rangle -\langle A_{0}^{\left(m\right)}B_{0}^{\left(\eta \right)}|V_{AB}^{\left(\eta \right)}|A_{0}^{\left(m\right)}B_{0}^{\left(\eta \right)}\rangle \right)\\ \;=E_{0}^{\left(m\right)}+\frac{1}{4\pi \varepsilon_{0}\varepsilon_{\text{eff}}}\sum_{I}\sum_{\eta ,J}\frac{\left(q_{I}^{\left(m\right)}(s,s)-q_{I}^{\left(m\right)}(0,0)\right)q_{J}^{\left(\eta \right)}(0,0)}{\left|{\text{R}}_{I}^{\left(m\right)}-{\text{R}}_{J}^{\left(\eta \right)}\right|}. \end{array}$$

Here, in Equation 9, $q_{I}^{\left(m\right)}(s,s)$ and $q_{I}^{\left(m\right)}(0,0)$ are the atomic partial charges of the dimer *m* in its excited and ground state, respectively. The values of these charges were obtained by fitting the electrostatic potential of the dimers *in vacuo*. $q_{J}^{\left(\eta \right)}(0,0)$ are the atomic partial charges of the environmental blocks. The partial charges of single chlorophylls and carotenoids in their ground state were determined by fitting the electrostatic potential as well. The calculation of the partial charges was performed using Gaussian 16 package (Frisch et al., 2016) at the TD-DFT/CAM-B3LYP level of theory with 6–31G(d) basis set and the Multiwfn software (Lu and Chen, 2011; Zhang and Lu, 2021). After assuming that single tailless chlorophylls of optimized geometry have very similar spatial structure, we chose two reference chlorophylls (*a*602 and *b*605) for the calculation of atomic partial charges. The values of partial charges of reference chlorophylls were used for all single chlorophylls (when chlorophylls were considered to be part of the environment) present in the pigment–protein complex (see **Supplementary Table S-I** in **Supplementary Material**). The calculation of the atomic partial charges of carotenoids was based on the original geometry of the molecules (see **Supplementary Table S-II** in **Supplementary Material**). While choosing non-optimized geometry might induce some error in the calculated partial charges, this was done to avoid possible geometry changes that would be hindered in the protein environment. Partial charges of the chlorophyll monomers (used for calculating energy shifts for monomers) are given in **Supplementary Tables S4–S33** in **Supplementary Material**. Partial charges for the chlorophyll dimers are given in **Supplementary Tables S34–S74** in **Supplementary Material**. The charges of hydrogen atoms for all the molecules (except amino acids) were set to 0. The atomic partial charges of amino acid molecules were taken from the CHARMM (Brooks et al., 2009) force field, including the charges of the hydrogen atoms [they were added using AMBER 2020 software (Case et al., 2020)].

### 2.3 Determining the protonation pattern of the protein

The largest part of the Lhca4 complex is the protein, consisting of 196 amino acid molecules. In order to simulate the environment properly, it is necessary to determine the protonation pattern of the protein. Besides the N-terminus and C-terminus of the protein chain, Lhca4 protein contains 6 types of titratable amino acids: asparatic acid (ASP), glutamatic acid (GLU), tyrosine (TYR), histidine (HIS), lysine (LYS) and arginine (ARG). The first three types are acidic titratable groups that are prone to releasing a proton to the environment and the latter three types are basic titratable groups that are likely to accept a proton from the environment. The corresponding Henderson–Hasselbalch equation for the acids is:


(10) $$\text{pH}=pK_{a}+\text{lg}\frac{\left[{\text{A}}^{-}\right]}{\left[\text{HA}\right]},$$

and for bases:


(11) $$\text{pH}=pK_{b}-\text{lg}\frac{\left[\text{B}{\text{H}}^{+}\right]}{\left[\text{B}\right]},$$

where the brackets denote the concentrations of the corresponding species at equilibrium. $pK_{b}=-\text{lg }K_{b}$ and $pK_{a}=-\text{lg }K_{a}$ are the logarithms of the equilibrium constants for the reaction of basic and acidic amino acids, respectively. If the equilibrium constant for the dissociation and association reactions as well as the pH of the environment are known, the most probable protonation pattern for every titratable group of the protein can be determined. In most cases, acidic titratable group is considered fully protonated if the difference between pH and the equilibrium constant is ≲ −2, and fully deprotonated if this difference is ≳ 2 (for basic titratable groups the rule is inverse).

## 3 Results

### 3.1 Estimating the protonation pattern and modelling the protein

The termini of the protein chain were modelled as follows: N-terminus was capped with a positively charged acetyl group and C-terminus was modelled with negatively charged COO^−^ group. For every titratable group present in the protein, the values of the equilibrium constant in neutral pH were estimated using PROPKA 3 tool (Olsson et al., 2011; Søndergaard et al., 2011) (see **Supplementary Table S-III** in **Supplementary Material**). There were 9 amino acids that had the absolute value of the difference between $pK_{a}$ and pH values less or equal to 2. These residues are ASP159, GLU95, GLU113, GLU145, GLU153, GLU202, HIS222, HIS236, and HIS242. Note that throughout the paper we use the numbering and the nomenclature of the PDB file 4XK8 (Qin et al., 2015) for the amino acids and chlorophylls, contrary to some of the literature, where often the nomenclature of the LHCII crystal structure (Liu et al., 2004) is used instead. Using the Henderson–Hasselbalch Equations 10 and 11 we determined that there are three amino acids that are in their non-standard protonation state (in neutral pH): GLU145, GLU153 and HIS242. These residues are considered protonated and their total charge is neutral for glutamatic acids and positive for histidine. The rest of the histidines were considered to be ϵ -tautomers. After setting the non-standard charges for the titratable groups mentioned above and the termini, the total charge of the Lhca4 protein chain is –5 (for full descriprion of the protein chain see **Supplementary Table S1** in **Supplementary Material**).

### 3.2 Energy shifts of chlorophyll monomers

**Figure 2:**
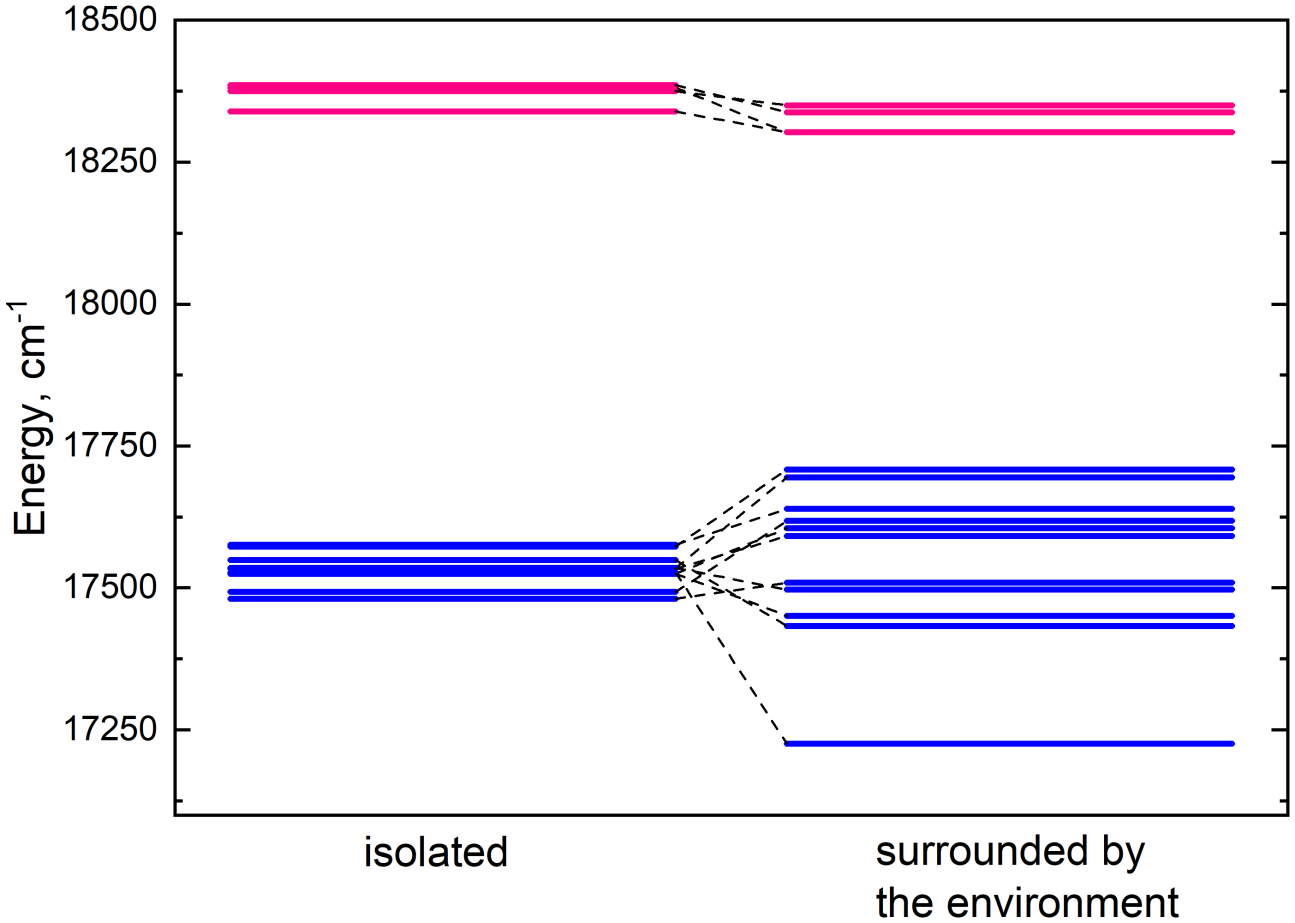
Energy level diagram of isolated (left column) monomer energies and monomer energies predetermined by the environment (right column). The energy levels of chlorophylls *a* and *b* are depicted in blue and pink respectively.

Lhca4 complex contains 15 chlorophyll monomers (11 chlorophylls *a* and 4 chlorophylls *b*). We calculated Q_y_ energies for these tailless monomers *in vacuo* as well as their Q_y_ energy shifts determined by the environmental blocks (other chlorophyll monomers, carotenoids, and the protein chain) using CDC method. The values are given in **Table 1**. The energy shifts are also depicted in **Figure 2**. The energy shifts are of the similar magnitude as was previously estimated for the PSI core using the same approach (Adolphs et al., 2010). The lowest –energy Chl *a* is *a*602, according to our calculations. Interestingly, in the previous modeling work on Lhca, where site energies were obtained by the fits of the linear optical spectra using the modified Redfield theory (Novoderezhkin et al., 2016), this was not the case. The current results indirectly supports the earlier assessment by some of us that the site energies obtained by fitting the optical lineshapes using the modified Redfield theory needs to be reevaluated (Gelzinis et al., 2015). It is also of note that the energy shifts for Chl *b* molecules are somewhat smaller than for Chl *a*, which should be related to the smaller difference values between the static dipole moments of the ground and the Q_y_ states. Interestingly, our calculations slightly overestimate the energy gap between the Chl *a* and Chl *b* pigments (at ∼ 800 cm^−1^), contrary to some of the previous work in literature, where the same functional and basis set was used, but the environmental effects were accounted for in a more advanced way (Jurinovich et al., 2015).

**Table 1:** Q_y_ energies of the tailless isolated chlorophyll monomers, energy shifts for these states calculated with CDC method $\left(\varepsilon_{\text{eff }}=\;1\right)$ , and their total Q_y_ energies in the Lhca4 complex.

Monomer	$E_{{\text{Q}}_{\text{y}}}$ , cm^-1^	$\Delta E_{{\text{Q}}_{\text{y}}}$ , cm^-1^	$E_{{\text{Q}}_{\text{y}}}+\Delta E_{{\text{Q}}_{\text{y}}}$ , cm^-1^	$\left|\mu_{1}-\mu_{0}\right|$ , D	$\left|\mu_{0\to 1}\right|$ , D	$\Delta E_{{\text{Q}}_{\text{y}}}^{N98H}$ , cm^-1^	$E_{{\text{Q}}_{\text{y}}}+\Delta E_{{\text{Q}}_{\text{y}}}^{N98H}$ , cm^-1^
*a*601	17576	64	17639	0.6062	5.1466	59	17635
*a*602	17527	-301	17226	0.6330	5.0764	-283	17244
*a*603	17481	28	17509	0.5303	5.1435	-151	17330
*a*604	17549	-117	17433	0.5986	5.1148	-114	17435
*a*608	17535	160	17695	0.5971	5.1646	169	17704
*a*609	17493	125	17618	0.6149	5.1338	120	17613
*a*610	17535	-38	17497	0.5936	5.1644	-37	17498
*a*611	17525	80	17605	0.6096	5.1453	81	17606
*a*612	17525	-74	17451	0.6049	5.0521	-70	17455
*a*613	17573	136	17709	0.8073	5.2488	138	17711
*a*614	17534	57	17591	0.5972	5.1900	53	17587
*b*605	18381	-43	18338	0.2503	4.1736	-40	18341
*b*606	18339	-36	18303	0.1315	4.1228	-39	18300
*b*607	18386	-48	18338	0.1844	4.0244	-43	18343
*b*615	18375	-25	18350	0.1548	3.9901	-26	18349

The difference between the static dipole moment values of the ground and the first excited states and the transition from the ground to the excited state dipole moment values for every monomer are given as well. The last two columns represent energy shifts and total Q_y_ energies in N98H mutant of Lhca4 complex (see Section 3.4).

### 3.3 Energy shifts of chlorophyll dimers

#### 3.3.1 Selected dimers and their CT states

We selected chlorophyll dimers that have the Mg–Mg distance less than 12Å. For each selected dimer, we performed quantum chemical calculations and compared static and transition from the ground to the excited state dipole moments, as well as the sum of Mulliken partial charges for each monomer of eight lowest excited states. Large values of static dipole moment and small values of transition dipole moment indicate the CT state (Premvardhan et al., 2008), and the sum of Mulliken partial charges for such state will be approximately equal to 1 for one pigment of the dimer and to –1 for another pigment (Voityuk, 2013). The results of these calculations for the dimer *a*610–*a*611 are given in **Table 2**. The values of the physical quantities mentioned above significantly differ for the fifth and sixth excited states. Therefore, these states are CT states.

**Table 2:** The comparison of the static (SDM) and transition (TDM) dipole moments for the first eight excited states of the dimer *a*610–*a*611.

State	*E*, cm^-1^	SDM, D		TDM, D	Mulliken charges	
		$\left|\mu_{n}\right|$	$\left|\mu_{n}-\mu_{0}\right|$	$\left|\mu_{0\to n}\right|$	a610	a611
*S*_0_	–	4.127	–	–	-0.02	0.02
*S*_1_	17180	4.075	0.103	7.814	-0.02	0.02
*S*_2_	17583	4.006	0.149	2.215	-0.02	0.02
*S*_3_	20406	3.570	1.123	1.161	-0.02	0.02
*S*_4_	20648	3.415	1.111	1.742	-0.02	0.02
*S*_5_	**24197**	**30.872**	**32.649**	**0.811**	**0.90**	**-0.90**
*S*_6_	**24762**	**36.266**	**34.022**	**0.280**	**-0.97**	**0.97**
*S*_7_	27504	2.888	1.528	0.656	0.01	-0.01
*S*_8_	28149	1.769	17.568	6.353	0.48	-0.48

The last two columns give the sum of Mulliken partial charges (in atomic units) for each pigment of the dimer. The values given in bold indicate the CT state.

The results for the rest of the selected dimers can be found in **Supplementary Material**. After analyzing the results for all 12 selected dimers, 19 low energy CT states were found in Lhca4 complex. It is crucial to stress that after performing calculations *in vacuo*, the Q_y_ energies of monomers (see **Table 1**) are far lower than the energies of CT states of dimers and it is only the environmental electrostatics effects that can make them comparable.

#### 3.3.2 Estimated protonation of the protein

**Figure 3:**
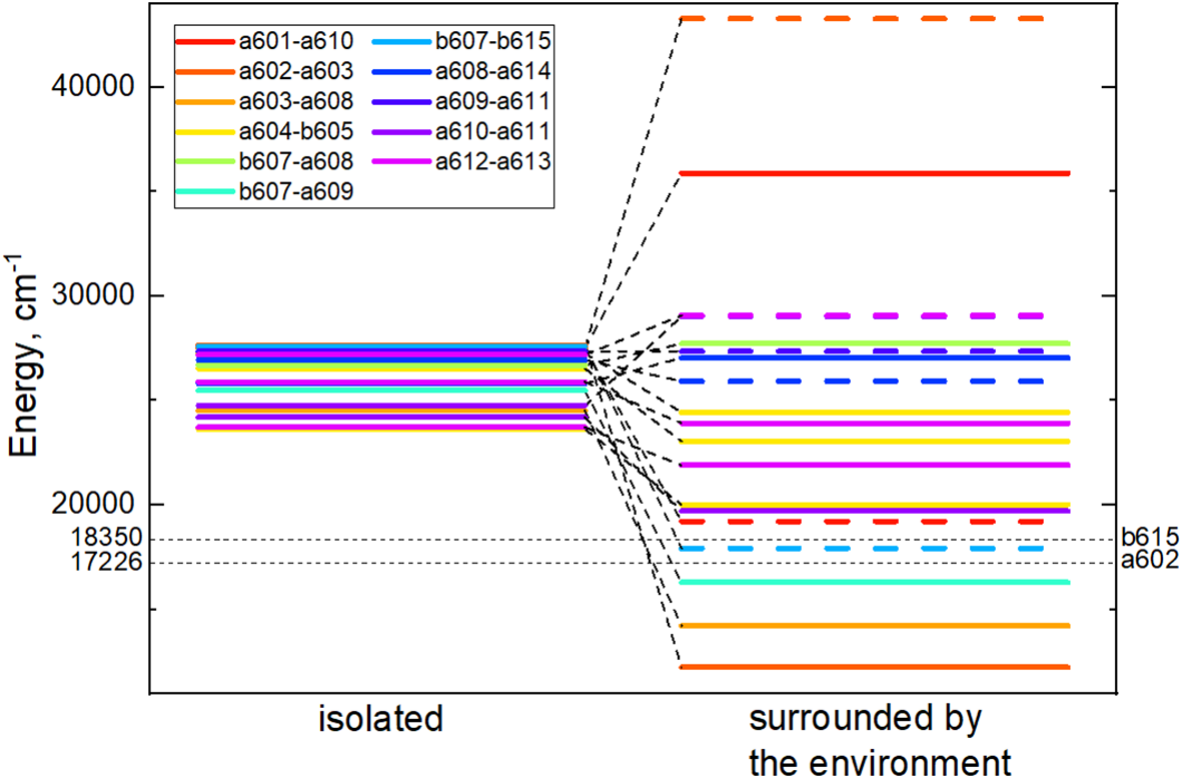
Energy level diagram of the isolated (left column) dimer CT state energies and CT energies predetermined by the environment (right column). In the right column, energies of the CT states, whose first pigment is negatively charged and the second one is positively charged, are given in dash. Cases, where the first pigment is positively charged and the second one is negatively charged, are given in solid lines. Black dashed lines mark the lowest and the highest monomer *Q_y_* energies predetermined by the environment.

For every CT state found in the Lhca4 complex we calculated the energy shift influenced by the environment. The protein is considered to be in the estimated protonation state where the total charge of the chain is –5. The environmental chlorophylls and carotenoids are in their ground state while their net charge is set to neutral. The energy shifts were calculated using the CDC method. The CT state energy values of the isolated dimers and energy shifts caused by the environment are given in **Table 3**. The energies of the CT states *in vacuo* and in the Lhca4 environment are compared in **Figure 3** as well.

**Table 3:** Energy shifts for the CT states determined using CDC method ( $\varepsilon_{\text{eff }}=\;1$ ).

No.	Dimer (Chl_1_–Chl_2_)	*R*, Å	*S*_CT_	*E*_0_, cm^–1^	Δ*E*, cm^–1^	*E*_0_ + Δ*E*, cm^–1^	Δ*E^N^* ^98^ *^H^*, cm^–1^	*E*_0_ + Δ*E^N^* ^98^ *^H^*, cm^–1^
1	*a*601–*a*610	11.75	*S*_5_(+–)	27505	8379	35884	8311	35816
2	*a*601–*a*610		*S*_6_(–+)	27505	–8281	19224	–8217	19288
3	*a*602–*a*603	11.87	*S*_5_( –+)	27505	15756	43261	16399	43904
4	*a*602–*a*603		*S*_6_(+–)	27666	–15428	**12238**	–16366	**11300**
5	*a*603–*a*608	8.93	*S*_6_(+–)	24521	–10292	**14229**	–8840	**15681**
6	*a*604–*b*605	8.67	*S*_5_(+–)	23633	–3598	20035	–3536	20097
7	*a*604–*b*605		*S*_8_(+–)	27263	–2831	24432	–2789	24474
8	*a*604–*b*605		*S*_6_(+–)	26537	–3488	23049	–3429	23108
9	*b*607–*a*608	10.46	*S*_5_(+–)	26698	1019	27717	276	26974
10	*b*607–*a*609	11.75	*S*_5_(+–)	25488	–9189	**16299**	–8990	**16498**
11	*b*607–*b*615	11.43	*S*_7_(–+)	27586	–9652	**17933**	–9398	**18188**
12	*a*608–*a*614	10.23	*S*_5_(+–)	25811	1231	27042	403	26214
13	*a*608–*a*614		*S*_6_(–+)	26940	–1012	25928	–254	26687
14	*a*609–*a*611	11.86	*S*_5_( –+)	27344	10	27354	299	27642
15	*a*610–*a*611	9.90	*S*_5_(+–)	24198	–4484	19714	–4460	19738
16	*a*610–*a*611		*S*_6_ (–+)	24762	4274	29036	4253	29015
17	*a*612–*a*613	9.40	*S*_5_(+–)	23714	–1805	21908	–1622	22092
18	*a*612–*a*613		*S*_6_ (+–)	25892	–1975	23916	–1812	24080
19	*a*612–*a*613		*S*_7_ (–+)	27182	1889	29071	1710	28892

The energies of the CT states of isolated pigments E_0_ and total site energies are given as well. Column R denotes the Mg–Mg distance between the pigments of the dimer. Column S_CT_ denotes the type of the CT state. The last two columns represent energy shifts and total CT state energies in N98H mutant of Lhca4 complex (see Section 3.4). The cases where monomer Q_y_ energy value exceeds the value of the CT state of the dimer are given in bold.

There are four dimers whose total energy of the CT state is lower than the *Q_y_* energy of monomers: *a*602–*a*603, *a*603–*a*608, *b*607–*a*609 and *b*607*–b*615. In principle, these CT states might contribute to the formation of the red-shifted peak in the experimental fluorescence spectra (Morosinotto et al., 2003; Wientjes and Croce, 2011). The former two dimers are more probable candidates, since our calculated electrostatic shifts were done by assuming $\varepsilon_{\text{eff }}=\;1$ , while in proteins this value is expected to be slightly higher.

The lists of environmental blocks that create the largest energy shifts for monomers (see **Table 4**) and dimers mentioned above (see **Table 5**) reveal that the protein influences both monomer Q_y_ energies and dimer CT energies more than remaining pigments. While looking at **Table 4**, one can notice that the majority of monomers listed are the ones that form the four dimers of interest and that the largest energy

**Table 4:** List of the environmental blocks that have the largest (≥ 80cm^−1^) influence on the monomer Q_y_ energies (in descending order).

Monomer	Env. block	Δ*E*, cm^−1^	Monomer	Env. block	Δ*E*, cm^−1^
a608	ARG156	244	a601	GLY54	118
*b*615	ARG155	203	*b*615	ASP169	-111
a609	ARG100	201	a610	LYS203	111
*b*607	ARG100	-193	a613	HIE236	111
a602	ARG209	165	a609	LYS201	98
*b*607	ARG156	-152	*b*606	SER138	-97
a602	GLU95	-148	a610	GLU199	-88
a611	LYS203	146	*b*606	GLU132	85
*b*607	ARG155	144	a613	PRO240	-84
a602	ASP77	-118			

**Table 5:** List of the environmental blocks that have the largest (≥ 4000cm^−1^) influence on the CT state energies of dimers *a*602–*a*603, *a*603–*a*608, *b*607–*a*609 and *b*607*–b*615 (in descending order).

Dimer	Env. block	Δ*E*, cm^−1^	Dimer	Env. block	Δ*E*, cm^−1^
*b*607*–b*615	**ASP169**	-10990	*b*607–*a*609	**ARG155**	-5931
*a*602–*a*603	**GLU95**	-10679	*a*603–*a*608	**ARG156**	-5354
*a*602–*a*603	**ARG209**	10505	*a*602–*a*603	**ASP77**	-5209
*b*607–*a*609	GLU204	-9338	*a*602–*a*603	ASP73	-4887
*b*607–*b*615	**ARG156**	-8191	*b*607–*a*609	ASP159	4823
*b*607–*a*609	**ARG156**	-7407	*b*607–*b*615	ASP159	4425
*b*607–*b*615	**ARG100**	-6861	*b*607–*a*609	LYS203	4041

The blocks that have large influence on corresponding monomer Q_y_ energies as well are given in bold.

shifts are calculated for these exact monomers (*a*602, *a*608, *a*609, *b*607 and *b*615). Amino acids that have significant influence on these monomers also have effect on the corresponding dimers that these monomers form (see **Table 5**). Amino acids that significantly influence both *a*602 monomer and *a*602–*a*603 dimer are GLU95, ASP77 and ARG209. For the dimer *a*603–*a*608 and monomer *a*608 the most significant impact comes from ARG156. Q_y_ energy of the monomer *b*607 and CT state energy of the dimer *b*607–*a*609 are

both shifted by ARG155 and ARG156 amino acids. The last dimer *b*607*–b*615 and monomer *b*615 are strongly influenced by ASP169, and the same dimer and monomer *b*607 are influenced by ARG156 and ARG100.

#### 3.3.3 Possible non-standard protonation of the protein

**Figure 4:**
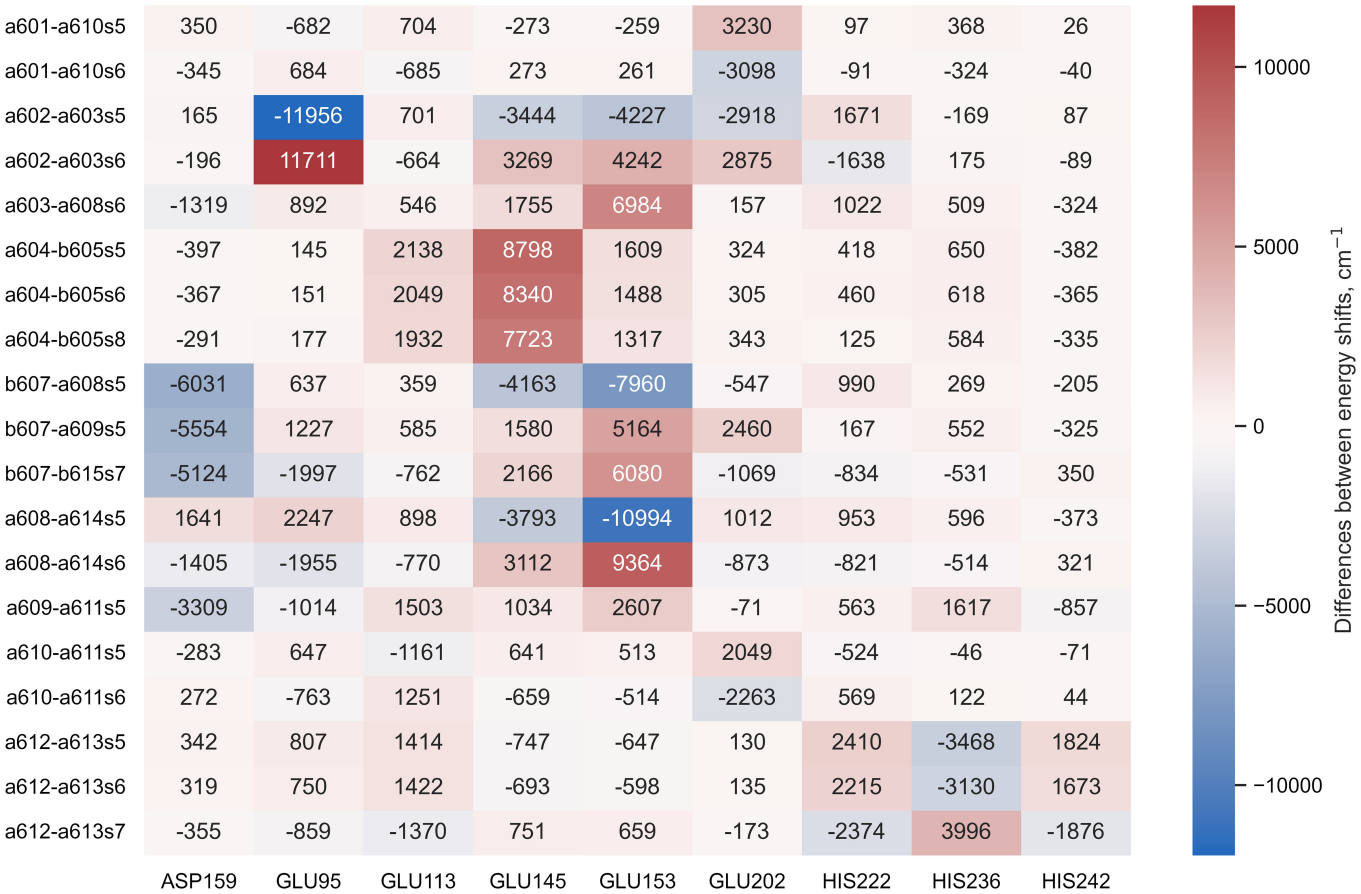
The difference between the energy shifts of the CT states, calculated in estimated protonation environment and the non-standard protonation state of the specific amino acid indicated at the bottom of each column.

**Figure 5:**
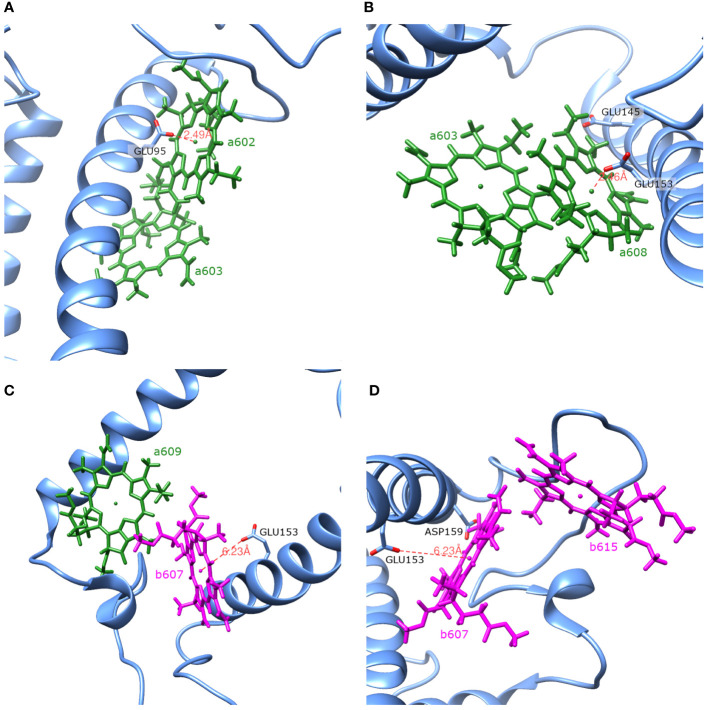
Spatial arrangement of the dimers a602–a603 **(A)**, a603–a608 **(B)**, b607–a609 **(C)** and b607–b615 **(D)** together with amino acids that have the largest influence on the Qy energies of the monomers and the CT energies of the dimers.

The Lhca4 protein chain contains nine amino acids with an absolute difference between their $pK_{a}$ (or $pK_{b}$ ) and pH values less than or equal to 2. In principle, these amino acids could easily gain/lose a proton under physiological conditions. We calculated the energy shifts for the CT states of selected dimers considering these various protonation patterns of the protein. There were nine distinct protonation states, with one of the nine amino acid residues exhibiting a protonation state opposite to the one estimated and all other residues exhibiting previously estimated protonation state. The differences in the CT state energy shifts calculated in estimated protonation environment and all non-standard protonation environments are given in the **Figure 4** (similar representation of the results in various protonation patterns for chlorophyll monomers is given in **Supplementary Figure S1** in **Supplementary Material**). The most significant energy shift differences after changing the protonation pattern of the protein are obtained for the dimers *a*602*–a*603, *a*603*–a*608, *a*604*–b*605, *b*607*–a*608, *b*607*–a*609, *b*607*–b*615, and *a*608*–a*614. Amino acids that are mostly responsible for these differences are protonated GLU95 (for the dimer *a*602*–a*603), standard GLU145 (for the dimer *a*604*–b*605), and standard GLU153 (for the dimers *a*603*–a*608, *b*607*–a*608, *b*607*–a*609, *b*607*–b*615, and *a*608*–a*614). Four dimers, whose CT state energies are lower than the monomer Q_y_ energies, together with amino acids, that create large energy shifts for these pigments, are shown in **Figure 5**.

### 3.4 N98H mutant

Apart from the WT environmetnt of Lhca4, we also checked whether the energy shifts for the CT states of the Chl dimers and the Q_y_ energies of the monomers would still lead to similar results in N98H mutant environment. In N98H mutant, the asparagine (ASN) in position 45 (counting from N-terminus) is substituted for histidine (HIS). We chose the most probable rotamer of histidine (probabililty ~36%) using UCSF Chimera software (Pettersen et al., 2004). The same methods were used for the mutated protein chain to find that the newly added amino acid is neutral *ε*-tautomer and thus the net charge of the protein remains unchanged.

Using the CDC method, energy shifts in the new environment for both monomers and dimers were calculated. The results are given in the last two columns of **Tables 1** and **3**, respectively. Compared to the results obtained in the environment of WT Lhca4, energies of the monomer Q_y_ and dimer CT states in N98H environment show no significant changes. After comparing the energies of the dimer CT states and the corresponding monomer Q_y_ states in N98H environment, it is clear that there are 4 CT states whose energy is lower than the Q_y_ energy of the monomers that form these dimers. These four states are the same as were obtained in WT Lhca4 environment.

## 4 Discussion

The presence of CT states in the Lhca4 complex has been suggested based on the significantly red-shifted peaks in the emission spectra. LHCII trimers, for instance, fluoresce at ∼ 680nm (Chmeliov et al., 2016), and almost the same fluorescence wavelength is observed for the monomeric CP29 complexes (Mascoli et al., 2019). For the light-harvesting antenna of PSI, the experimental investigations are hindered by the fact that Lhca1–4 monomers cannot be isolated. Nonetheless, both Lhca1/4 heterodimer (Wientjes and Croce, 2011), and reconstituted Lhca4 complexes (Morosinotto et al., 2003) show red-shifted fluorescence, with peaks at ∼ 730nm. While initially this was thought to be related to excitonic interaction (Morosinotto et al., 2003), it was soon proposed that CT states should play a role (Ihalainen et al., 2005), which was confirmed later by Stark absorption measurements (Romero et al., 2009). Another point to consider is that Lhca4 complexes might exist in multiple conformations. This is supported by single-molecule spectroscopy fluorescence measurements (Krüger et al., 2011) and by time-resolved fluorescence measurements (Wientjes et al., 2011). All this implies that the spectroscopic features of Lhca4 might be influenced by more than one CT state. Therefore, in this work we have performed a systematic quantum-chemical investigation of the possible CT states, paying particular attention to the electrostatic environment-induced shifts of the CT state energies. Below we will discuss the implications of our work in more detail.

We first note that we have found 4 possible CT states that, in principle, could contribute to the experimental fluorescence signal: *a*602^+^–*a*603^−^, *a*603^+^–*a*608^−^, *b*607^−^–*a*609^+^, and *b*607^−^–*b*615^+^. The calculated energies of the latter two states are reasonably close to the Q_y_ manifold, as the mean Q_y_ energy for Chls *a* is 17543cm^−1^ and the lowest energy, corresponding to *a*602, is 17226cm^−1^. For simplicity, all calculations in this work were done using $\varepsilon_{\text{eff }}=\;1$ . Note that $\varepsilon_{\text{eff}}$ is an effective scaling factor, accounting for both the dielectric screening of the protein environment and possible systematic errors in the evaluation of the atomic partial charges. Previous theoretical work often assumed $\varepsilon_{\text{eff }}=\;1.5$ ; therefore, after performing calculations with $\varepsilon_{\text{eff }}=\;1$ , we have also checked the effect of its variation within the interval between 1 and 1.5.Assuming this value, the estimated energies of the latter two CT states would be much higher than the Q_y_ manifold, while the energies of the former two states would become $E_{a602^{+}-a603^{-}}=\;17381\text{c}{\text{m}}^{-1}$ and $E_{a603^{+}-a608^{-}}=17659\text{ c}{\text{m}}^{-1}$ , which would put them in the middle of the Q_y_ manifold. A slightly smaller value of $\varepsilon_{\text{eff }}=\;1.3$ would result in $E_{a602^{+}-a603^{-}}=15799\text{ c}{\text{m}}^{-1}$ and $E_{a603^{+}-a608^{-}}=16604\text{ c}{\text{m}}^{-1}\;$ , and the corresponding energy gap to the Q_y_ manifold would then become comparable to the red-shifted fluorescence peak. We thus propose that both of these states could contribute to the experimental fluorescence signal, although fine-tuning of the $\varepsilon_{\text{eff}}$ value in the future detailed theoretical modeling is needed to precisely describe the whole spectrum. The latter two (energetically higher) CT states will hardly contribute to the red-shifted fluorescence, but their presence might still be needed to correctly reproduce all the features of the experimental data, especially those observed in the Stark measurements (Romero et al., 2009).

Interestingly, previously only the *a*603–*a*608 dimer (note that it is often called *a*603–*a*609 dimer due to the prevalent usage of the LHCII nomenclature) was considered to be the origin of the relevant CT state in both experimental considerations (Ihalainen et al., 2005; Romero et al., 2009) and theoretical calculations (Novoderezhkin et al., 2016; Sláma et al., 2023). One of the reasons for that is that the Mg–Mg distance between Chls *a*603 and *a*608 is much shorter than between *a*602 and *a*603 (see **Table 3**). Clearly, a short enough distance between two pigments is a necessary, but not sufficient condition for a spectroscopically active CT state to occur therein. Indeed, even for the Lhca4 complex, the shortest distance between the two pigments corresponds to the *a*604–*b*605 dimer, yet no low-lying CT state is present there. At this point it is also worthwhile to point out a recent quantum-chemical work on the PSII reaction center (Sirohiwal et al., 2020b), where it was shown that no low-energy CT states could be identified between the so-called special pair pigments P_D1_ and P_D2_, despite a very small spatial separation. Clearly, the environmental effects on the CT state energetics are of the paramount importance.

There is another argument that is often employed to claim that the *a*603–*a*608 dimer is the spatial origin of the CT states in the Lhca4 complex. It comes from site-directed mutagenesis. The amino acid coordinating the *a*603 pigment can be changed from ASN to HIS (N98H mutant in our notation, often called N47H elsewhere in literature). It was demonstrated that for this mutant the fluorescence spectrum of the reconstituted Lhca4 loses the red-shifted peak almost entirely (Morosinotto et al., 2003). Very recently, a partial loss of the red-shifted fluorescence in this mutant was also demonstrated for intact PSI–LHCI supercomplexes (Li et al., 2023). In addition, recent advanced quantum-chemical calculations also demonstrated the loss of the red-shifted fluorescence for this Lhca4 mutant (Sláma et al., 2023). We have performed calculations for this mutant as well, but our results do not show any huge differences for the CT state energies. At first glance, this seems to make our results to be at odds with the literature data. That is not the case, however. What our calculations show, is that solely replacing ASN98 to HIS98 while keeping the geometry of the nearby Chls the same, cannot account for huge energy shifts, neither for *a*602^+^–*a*603^−^, nor for *a*603^+^–*a*608^−^ dimers. Therefore, protein electrostatics is not the main factor in this case. Indeed, Sláma et al. (2023) demonstrated that this mutation induces spatial shifts for *a*603 pigment. It is these shifts that are the reason for different CT state energetics. We must stress that changes in the spatial position of Chl *a*603 would also induce significant changes in the *a*602^+^–*a*603^−^ energetics. Thus, it might be that the N98H lacks both of these CT states, and this is the reason for the disappearance of the red-shifted fluorescence.

We must also highlight that our suggestion that the *a*602^+^–*a*603^−^ state also contributes to the experimentally observable red-shifted fluorescence of the Lhca4 complex is indirectly supported by experimental evidence. Morosinotto et al. (2005) investigated several Lhca4 mutants, one of them being E95V/R209L double mutant (E44V/R158L in the original notation of Ref (Morosinotto et al., 2005)). GLU95 coordinates *a*602 and ARG209 is quite close to it, thus these mutations should affect the geometry of this pigment. Indeed, this mutant no longer fluoresces at ~730 nm, though fluorescence at ~ 700 nm remains (Morosinotto et al., 2005). Thus, it is very probable that *a*602 is involved in one of the spectroscopically active CT states, which our work shows to be *a*602^+^–*a*603^−^.

Let us now discuss the protonation pattern of the Lhca4 protein. According to our estimations, there are 31 charged amino acids at neutral pH, not counting the N- and C-terminus. Almost all the charged amino acids are quite close to the stromal or lumenal surfaces. The majority of the charged amino acids are on the stromal side of the membrane. Two of the three amino acids not exhibiting the standard protonation pattern (GLU145 and GLU153) are neutral and are located deeper inside the protein, while HIS242 is positively charged and located near the lumenal surface. Of all the charged amino acids, only the guanidino group of ARG156 quite far from the stromal surface and oriented towards the inside of the protein. We thus find that the estimated protonation should be reliable since both methods and software used are time-tested and proven to be credible and that the resulting protonation pattern does not contain any unexpected features.

Under physiological conditions, the protonation pattern of the amino acids results from a statistical process, and some of the amino acids could be easily (de)protonated. This, in turn, could shift the energy levels of the chlorophyll excited states, leading to different conformational states of the whole pigment–protein complex. Indeed, for Lhca4 the single-molecule spectroscopy measurements (Krüger et al., 2011) and time-resolved fluorescence measurements (Wientjes et al., 2011) suggest the coexistence of a few conformational states. We have investigated the possible non-standard protonation states of the Lhca4 complex by considering amino acids with an absolute difference between their $pK_{a}$ and pH values not exceeding 2. The resulting energy shifts for the Chl Q_y_ states are presented in **Supplementary Figure S1**, while the shifts for the identified CT states are presented in **Figure 4**. We will now discuss these possibilities in more detail.

Our identification of the easily protonable amino acids was based on the corresponding $pK_{a}$ values. Some additional consideration should be applied, however, because some of these amino acids are coordinating the pigments in the Lhca4 complex. GLU95 coordinates Chl *a*602, GLU153 coordinates Chl *a*608, and HIS236 coordinates Chl *a*613. Thus we hold that it is rather unlikely for these amino acids to change their protonation state, since that might affect the surrounding geometry. GLU145 does not directly coordinate the Mg atom of any Chl, yet it coordinates the water molecule that coordinates Chl *b*605 and it also coordinates the C7-formyl of Chl *b*606, thus it is also unlikely for this amino acid to change its protonation state. GLU113 and HIS242 are both on the periphery of the complex on the lumenal side. According to our calculations, the change of the protonation state of these amino acids would not result in any significant energy shifts for both the Chl Q_y_ states and the CT states that could contribute to the red emission. ASP159 is on the stromal side of the complex, and if it were to acquire a proton, this would result in significant decrease of the *b*607^−^–*a*609^+^ and *b*607^−^–*b*615^+^ state energies, making them comparable to the energies of *a*602^+^–*a*603^−^ and *a*603^+^–*a*608^−^. This could result in an increase of the red emission. On the other hand, the effects of the protonation state to the Chl Q_y_ state energies are minor. GLU202 and HIS222 are both on the periphery of the Lhca4 complex (on the stromal and lumenal sides, respectively). The changes of their protonation states would result in either higher (in case of GLU202) or lower (in case of HIS222) energy of the *a*602^+^–*a*603^−^ state, which could affect the low energy emission in the fluorescence spectra. Regarding the Chl Q_y_ states, all the calculated energy shifts are less than 100cm^−1^, with the largest effect being an increase of *b*606 Q_y_ energy by 84cm^−1^ in case of protonated HIS202.

There is one other issue that should be addressed. In the present work, similarly to many other works in literature (Frankcombe, 2015; Kavanagh et al., 2020; Sirohiwal et al., 2020b), we have considered chlorophyll dimers as a large supermolecule, and the identified CT states are then the eigenstates of this supersystem. The obtained CT states are thus not pure, but mixed (at least partially) with the excitonic states. This approach allows us to easily identify the energetically relevant CT states, but it must be kept in mind, that if the mixing with bright states is vanishingly small, such states would not receive oscillator strength; hence, these CT states would not be visible spectroscopically. Future studies thus should also consider the degree of mixing of pure CT states with pure site-basis excitations, which could provide the couplings of the CT states to the bright states that could then in principle be used to model the population transfer between all possible excited states. Such couplings could be obtained via the diabatization procedure, as in Ref (Nottoli et al., 2018).

## 5 Conclusion

In this work, we have performed a systematic study of possible CT states in the Lhca4 complex. In vacuum, all the CT states have energies much higher than the Q_y_ states of the pigments. However, when the electrostatic effects of the protein environment are taken into account, the energies of some of CT states drop down significantly. Based on our calculations, we propose that in addition to the *a*603^+^–*a*608^−^ CT state, also the *a*602^+^–*a*603^−^ state contributes to the far-red fluorescence signals of the Lhca4 complexes, which is supported by the experimental work on site-directed mutants. We have also investigated the possible protonation patterns of the titrable amino acids, and found that changes in protonation states of ASP159, GLU202 and HIS222 could affect the low energy emission. Future work should investigate the couplings between the pure CT states and molecular excited states, which would allow one to estimate the CT state populations.

## Data availability statement

The original contributions presented in the study are included in the article/**Supplementary Material**. Further inquiries can be directed to the corresponding author.

## Author contributions

GR: Formal analysis, Investigation, Methodology, Software, Visualization, Writing – original draft. AG: Conceptualization, Investigation, Methodology, Writing – original draft. BR: Conceptualization, Funding acquisition, Supervision, Writing – original draft. LV: Conceptualization, Methodology, Supervision, Writing – review & editing. JC: Conceptualization, Funding acquisition, Investigation, Methodology, Project administration, Writing – review & editing.
