# Supplementary material for: Environment-dependent chlorophyll–chlorophyll charge transfer states in Lhca4 pigment–protein complex
Source: Front Plant Sci. 2024 Aug 7;15:1412750. doi: 10.3389/fpls.2024.1412750 (PMC11335733; doi:10.3389/fpls.2024.1412750)
Supplement: Supplementary file 1 [file DataSheet_1.pdf]

Environment-dependent chlorophyll–chlorophyll charge transfer states in Lhca4 pigment–protein complex

Gabrielė Rankelytė[1,2], Andrius Gelzinis[1,2], Bruno Robert[3], Leonas Valkunas[2] and Jevgenij Chmeliov[1,2,*]

[1]*Institute of Chemical Physics, Faculty of Physics, Vilnius University, Vilnius, Lithuania*
[2]*Department of Molecular Compound Physics, Center for Physical Sciences and Technology, Vilnius, Lithuania*
[3]*Université Paris-Saclay, CEA, CNRS, Institute for Integrative Biology of the Cell, Gif-sur-Yvette, France*

Supplementary Material

1 Partial charges of the pigments

Table S-I. Partial charges of reference chlorophylls *a* and *b* in their ground state based on fit of the electrostatic potential. The charges of hydrogen atoms are set to 0.

Chl *a*602				Chl *b*605			
Atom	Charge	Atom	Charge	Atom	Charge	Atom	Charge
MG	0.862	C1	0.265	MG	0.813	C1	0.280
NA	-0.453	CMA	-0.021	NA	-0.333	CMA	-0.012
C1A	0.249	C2B	-0.057	C1A	0.177	C2B	-0.050
CHA	-0.308	C3B	-0.119	CHA	-0.365	C3B	-0.146
C4D	0.261	CAB	0.011	C4D	0.322	CAB	0.046
ND	-0.421	CBB	0.038	ND	-0.435	CBB	0.020
C1D	-0.007	CMB	0.119	C1D	0.009	CMB	0.103
CHD	0.080	C2C	-0.078	CHD	0.069	C2C	-0.149
C4C	0.094	C3C	-0.122	C4C	0.030	C3C	-0.006
NC	-0.466	CAC	0.096	NC	-0.330	CAC	0.102
C1C	0.187	CBC	0.002	C1C	0.060	CBC	0.003
CHC	-0.079	CMC	0.096	CHC	-0.016	CMC	0.455
C4B	0.198	C2D	0.025	C4B	0.202	OMC	-0.454
NB	-0.470	C3D	-0.207	NB	-0.506	C2D	0.041
C1B	0.167	CAD	0.436	C1B	0.240	C3D	-0.237
CHB	-0.075	OBD	-0.460	CHB	-0.135	CAD	0.436
C4A	0.056	CBD	0.088	C4A	0.025	OBD	-0.451
C3A	0.179	CGD	0.623	C3A	0.186	CBD	0.135
C2A	0.025	O1D	-0.502	C2A	0.138	CGD	0.662
CAA	0.006	O2D	-0.451	CAA	-0.051	O1D	-0.547
CBA	0.026	CED	0.303	CBA	-0.047	O2D	-0.447
CGA	0.671	CMD	0.110	CGA	0.709	CED	0.302
OA1	-0.525			O1A	-0.521	CMD	0.117
O2A	-0.453			O2A	-0.446		

Table S-II. Partial charges of carotenoids LUT, XAT and BCR in their ground state based on fit of the electrostatic potential. The charges of hydrogen atoms are set to 0.

LUT				XAT				BCR			
Atom	Charge	Atom	Charge	Atom	Charge	Atom	Charge	Atom	Charge	Atom	Charge
C1	0.381	C25	-0.816	C1	0.764	C24	-0.256	C1	0.388	C22	-0.318
C2	-0.274	C26	0.704	C2	-0.414	C25	0.803	C2	-0.051	C23	0.127
C3	0.259	C27	0.513	C3	0.342	C26	-0.629	C3	-0.039	C24	0.027
C4	0.238	C28	-0.285	C4	0.116	C27	0.496	C4	0.050	C25	-0.193
C5	-0.243	C29	-0.330	C5	0.098	C28	-0.045	C5	0.014	C26	0.018
C6	-0.100	C30	0.300	C6	-0.155	C29	-0.252	C6	-0.204	C27	0.042
C7	-0.004	C31	-0.209	C7	-0.048	C30	0.219	C7	0.017	C28	-0.027
C8	0.100	C32	0.133	C8	0.145	C31	-0.191	C8	0.137	C29	-0.060
C9	-0.338	C33	-0.364	C9	-0.360	C32	0.169	C9	-0.317	C30	0.363
C10	0.138	C34	0.199	C10	0.138	C33	-0.392	C10	0.108	C35	0.205
C11	-0.138	C35	-0.127	C11	-0.116	C34	0.205	C11	-0.125	C36	0.211
C12	0.156	C36	-0.393	C12	0.146	C35	-0.127	C33	-0.005	C37	0.157
C13	-0.397	C37	-0.330	C13	-0.400	C36	-0.097	C31	-0.070	C38	-0.016
C14	0.188	C38	0.040	C14	0.197	C37	-0.121	C32	-0.137	C39	-0.093
C15	-0.060	C39	0.155	C15	-0.059	C38	-0.462	C34	0.165	C40	-0.100
C16	-0.043	C40	0.237	C16	-0.127	C39	0.109	C12	0.141		
C17	-0.075	O23	-0.075	C17	-0.164	C40	0.240	C13	-0.363		
C18	0.044			C18	0.009	O23	-0.144	C14	0.185		
C19	0.184			C19	0.197	O24	-0.144	C15	-0.091		
C20	0.225			C20	0.219			C16	-0.099		
O3	-0.207			O3	-0.207			C17	0.200		
C21	0.662			O4	-0.293			C18	-0.387		
C22	-0.724			C21	0.489			C19	0.140		
C23	0.323			C22	-0.275			C20	-0.105		
C24	0.352			C23	0.375			C21	0.104		

2 pKa values of titratable amino acids

Table S-III. pKa values of titratable amino acids in Lhca4 protein chain. The values were obtained considering neutral pH using PROPKA 3 tool.

Amino acid	ID	Estimated pKa	Amino acid	ID	Estimated pKa
ASP	73	4.44	HIS	222	5.01
ASP	77	2.83	HIS	236	5.35
ASP	84	4.02	HIS	242	7.1
ASP	159	6.69	TYR	65	12.83
ASP	169	1.62	TYR	127	11.62
ASP	231	4.09	TYR	134	11.83
ASP	239	3.39	TYR	151	10.65
GLU	55	4.59	TYR	175	10.28
GLU	83	4.87	TYR	184	14.06
GLU	86	4.66	LYS	125	10.52
GLU	95	6.78	LYS	131	10.33
GLU	113	5.79	LYS	161	10.06
GLU	132	4.72	LYS	173	10.39
GLU	133	4.68	LYS	201	9.52
GLU	145	8.69	LYS	203	9.93
GLU	153	7.14	LYS	227	10.61
GLU	181	4.41	ARG	89	12.16
GLU	199	4.29	ARG	100	12.6
GLU	202	5.78	ARG	155	11.54
GLU	204	-0.04	ARG	156	10.7
HIS	150	4.44	ARG	209	11.07

3 Results of the excited state calculations of selected dimers

Table S-IV. The comparison of static and transition dipole moments for the first eight excited states of selected dimers. The last two columns give the sum of Mulliken partial charges for each pigment of the dimer.

State	E , cm^{-1}	SDM, D		TDM, D	Mulliken charges	
		$\lvert\mu_n\rvert$	$\lvert\mu_n - \mu_0\rvert$	$\lvert\mu_{0\to n}\rvert$	1st mol.	2nd mol.
**a601–a610**						
S_0	-	0.443	-	-	0.00	0.00
S_1	17503	0.562	0.275	2.214	0.00	0.00
S_2	17583	0.705	0.321	6.683	0.00	0.00
S_3	20648	1.552	1.183	1.713	0.00	0.00
S_4	20729	1.018	1.240	1.698	0.00	0.00
S_5	27504	53.026	53.400	0.000	1.00	-1.00
S_6	27504	54.044	53.670	0.000	-1.00	1.00
S_7	28149	1.597	1.202	9.122	0.00	0.00
S_8	28230	0.881	1.204	6.494	0.00	0.00
**a602–a603**						
S_0	-	8.520	-	-	0.00	0.00
S_1	17422	8.419	0.366	6.409	0.00	0.00
S_2	17503	8.327	0.567	3.935	0.00	0.00
S_3	20568	7.466	1.250	1.587	0.00	0.00
S_4	20729	7.277	1.314	1.707	0.00	0.00
S_5	27504	56.920	51.809	0.312	-0.98	0.98
S_6	27666	47.017	51.081	0.774	0.95	-0.95
S_7	27988	6.654	2.498	3.571	0.03	-0.03
S_8	28149	7.180	1.518	10.732	0.00	0.00
**a603–a608**						
S_0	-	6.218	-	-	0.11	-0.11
S_1	16696	5.636	0.895	7.658	0.08	-0.08
S_2	17341	6.124	0.106	3.067	0.11	-0.11
S_3	19519	5.012	1.474	1.169	0.07	-0.07
S_4	20487	5.596	1.060	1.620	0.11	-0.11
S_5	21778	19.947	23.247	0.980	-0.66	0.66
S_6	24520	29.162	24.829	1.490	0.91	-0.91
S_7	25084	16.282	19.630	2.293	-0.54	0.54
S_8	27101	6.321	1.650	1.114	0.14	-0.14
**a604–b605**						
S_0	-	12.890	-	-	-0.01	0.01
S_1	17503	12.528	0.573	4.667	-0.01	0.01
S_2	18390	12.597	0.293	4.127	-0.01	0.01
S_3	20729	12.024	1.125	1.420	-0.01	0.01
S_4	21132	12.694	1.391	1.202	-0.01	0.01
S_5	23633	34.366	35.365	0.391	0.97	-0.97

Table S-IV (continued)

State	E, cm^{-1}	SDM, D		TDM, D	Mulliken charges	
		$\lvert\mu_n\rvert$	$\lvert\mu_n-\mu_0\rvert$	$\lvert\mu_{0\to n}\rvert$	1st mol.	2nd mol.
S_6	26536	31.989	32.860	1.351	0.94	-0.94
S_7	26940	13.765	1.578	8.423	0.02	-0.02
S_8	27262	33.853	33.902	0.776	0.95	-0.95
**b605–b606**						
S_0	-	4.648	-	-	-0.01	0.01
S_1	18229	4.777	0.161	3.427	-0.01	0.01
S_2	18471	4.831	0.278	4.328	-0.01	0.01
S_3	20810	3.317	1.640	1.359	-0.02	0.02
S_4	21213	5.095	1.569	0.673	0.00	0.00
S_5	25084	4.173	2.309	13.193	-0.06	0.06
S_6	26375	12.032	9.166	0.801	0.24	-0.24
S_7	26536	23.336	24.309	1.970	-0.67	0.67
S_8	26859	13.969	11.850	2.202	0.32	-0.32
**b607–a608**						
S_0	-	6.061	-	-	0.00	0.00
S_1	17503	5.626	0.570	4.635	0.00	0.00
S_2	18390	6.121	0.127	4.131	0.00	0.00
S_3	20648	5.146	1.166	1.573	0.00	0.00
S_4	21213	5.884	1.208	0.617	0.00	0.00
S_5	26698	48.905	48.600	0.000	-1.00	-1.00
S_6	26778	6.933	2.021	7.150	0.00	0.00
S_7	28149	6.193	1.174	7.214	0.00	0.00
S_8	28149	6.405	1.215	8.389	0.00	0.00
**b607–a609**						
S_0	-	5.018	-	-	0.03	-0.03
S_1	17422	4.640	0.706	5.714	0.03	-0.03
S_2	18390	5.079	0.125	3.955	0.03	-0.03
S_3	20648	4.517	1.188	1.638	0.02	-0.02
S_4	21213	6.112	1.288	0.704	0.03	-0.03
S_5	25488	48.511	45.570	0.116	-0.96	0.96
S_6	26778	6.958	1.970	7.651	0.03	-0.03
S_7	27988	5.581	0.829	8.737	0.02	-0.02
S_8	28230	7.307	2.317	8.446	0.03	-0.03
**b607–b615**						
S_0	-	6.111	-	-	0.01	-0.01
S_1	18309	6.058	0.118	3.913	0.01	-0.01
S_2	18471	6.474	0.371	4.261	0.01	-0.01
S_3	21132	7.126	1.326	0.700	0.01	-0.01
S_4	21294	6.150	1.267	0.474	0.02	-0.02
S_5	26214	7.374	1.749	8.406	0.02	-0.02

Table S-IV (continued)

State	E, cm^{-1}	SDM, D		TDM, D	Mulliken charges	
		$\|\mu_n\|$	$\|\mu_n - \mu_0\|$	$\|\mu_{0\to n}\|$	1st mol.	2nd mol.
S_6	26859	8.188	2.185	9.580	0.01	-0.01
S_7	27585	51.739	46.899	0.361	-0.97	0.97
S_8	27907	8.098	2.732	6.406	0.01	-0.02
**a608–a614**						
S_0	-	8.658	-	-	-0.01	0.01
S_1	17503	8.354	0.427	6.276	-0.01	0.01
S_2	17583	8.282	0.389	4.298	-0.01	0.01
S_3	20648	9.309	1.137	1.710	-0.01	0.01
S_4	20729	7.968	1.021	1.359	-0.01	0.01
S_5	25810	40.325	44.100	0.095	0.96	-0.96
S_6	26940	42.996	37.957	2.828	-0.88	0.88
S_7	27666	10.144	2.677	8.312	-0.05	0.05
S_8	28311	9.800	2.401	8.411	-0.05	0.05
**a609–a611**						
S_0	-	6.498	-	-	0.00	0.00
S_1	17422	6.405	0.618	6.238	0.01	-0.01
S_2	17503	6.107	0.485	4.127	0.00	0.00
S_3	20648	5.551	1.233	1.924	0.00	-0.01
S_4	20648	5.569	1.269	1.366	0.00	-0.01
S_5	27343	55.094	52.312	0.185	-0.98	0.98
S_6	27907	27.951	29.833	2.515	0.55	-0.55
S_7	27988	22.504	24.262	2.608	0.45	-0.45
S_8	28149	5.587	1.447	10.936	0.01	-0.01
**a612–a613**						
S_0	-	6.907	-	-	0.01	-0.01
S_1	17341	6.397	0.544	5.379	0.01	-0.01
S_2	17503	6.739	0.664	5.248	0.01	-0.01
S_3	20406	6.740	1.270	1.689	0.01	-0.01
S_4	20729	6.121	1.097	1.542	0.01	-0.01
S_5	23713	32.877	34.267	0.794	0.94	-0.94
S_6	25891	30.059	31.244	2.012	0.90	-0.90
S_7	27182	36.661	34.112	1.074	-0.91	0.91
S_8	27907	6.918	1.660	8.862	0.00	0.00

4 Monomer energy shift changes in various non-standard protonation patterns

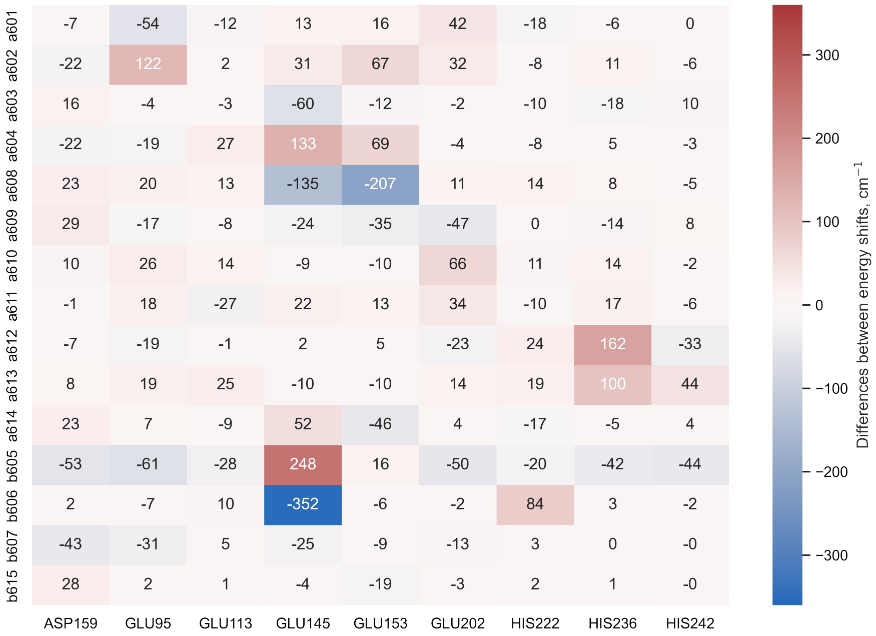

Figure S1. The difference of energy shifts calculated in estimated protonation environment and all non-standard protonation environments for all Qy energies of monomers.

5 Other Supplementary data included in the *Supplementary_Material.xlsx* file

Worksheet	Data presented
Table S1	Net charges of the protein amino acids
Table S2	Atomic coordinates and partial charges of Chl *a*602, used as a reference for all Chl *a* pigments
Table S3	Atomic coordinates and partial charges of Chl *b*605, used as a reference for all Chl *b* pigments
Table S4	Atomic coordinates and partial charges of Chl *a*601 in its ground state.
Table S5	Atomic coordinates and partial charges of Chl *a*601 in its first excited state state.
Table S6	Atomic coordinates and partial charges of Chl *a*602 in its ground state.
Table S7	Atomic coordinates and partial charges of Chl *a*602 in its first excited state state.
Table S8	Atomic coordinates and partial charges of Chl *a*603 in its ground state.
Table S9	Atomic coordinates and partial charges of Chl *a*603 in its first excited state state.
Table S10	Atomic coordinates and partial charges of Chl *a*604 in its ground state.
Table S11	Atomic coordinates and partial charges of Chl *a*604 in its first excited state state.

Worksheet	Data presented
Table S12	Atomic coordinates and partial charges of Chl *a*608 in its ground state.
Table S13	Atomic coordinates and partial charges of Chl *a*608 in its first excited state state.
Table S14	Atomic coordinates and partial charges of Chl *a*609 in its ground state.
Table S15	Atomic coordinates and partial charges of Chl *a*609 in its first excited state state.
Table S16	Atomic coordinates and partial charges of Chl *a*610 in its ground state.
Table S17	Atomic coordinates and partial charges of Chl *a*610 in its first excited state state.
Table S18	Atomic coordinates and partial charges of Chl *a*611 in its ground state.
Table S19	Atomic coordinates and partial charges of Chl *a*611 in its first excited state state.
Table S20	Atomic coordinates and partial charges of Chl *a*612 in its ground state.
Table S21	Atomic coordinates and partial charges of Chl *a*612 in its first excited state state.
Table S22	Atomic coordinates and partial charges of Chl *a*613 in its ground state.
Table S23	Atomic coordinates and partial charges of Chl *a*613 in its first excited state state.
Table S24	Atomic coordinates and partial charges of Chl *a*614 in its ground state.
Table S25	Atomic coordinates and partial charges of Chl *a*614 in its first excited state state.
Table S26	Atomic coordinates and partial charges of Chl *b*605 in its ground state.
Table S27	Atomic coordinates and partial charges of Chl *b*605 in its first excited state state.
Table S28	Atomic coordinates and partial charges of Chl *b*606 in its ground state.
Table S29	Atomic coordinates and partial charges of Chl *b*606 in its first excited state state.
Table S30	Atomic coordinates and partial charges of Chl *b*607 in its ground state.
Table S31	Atomic coordinates and partial charges of Chl *b*607 in its first excited state state.
Table S32	Atomic coordinates and partial charges of Chl *b*615 in its ground state.
Table S33	Atomic coordinates and partial charges of Chl *b*615 in its first excited state state.
Table S34	Atomic coordinates and partial charges of dimer *a*601-*a*610 in its ground state.
Table S35	Atomic coordinates and partial charges of dimer *a*601-*a*610 in its first excited state.
Table S36	Atomic coordinates and partial charges of dimer *a*601-*a*610 in its fifth excited (CT) state.
Table S37	Atomic coordinates and partial charges of dimer *a*601-*a*610 in its sixth excited (CT) state.
Table S38	Atomic coordinates and partial charges of dimer *a*602-*a*603 in its ground state.
Table S39	Atomic coordinates and partial charges of dimer *a*602-*a*603 in its first excited state.
Table S40	Atomic coordinates and partial charges of dimer *a*602-*a*603 in its fifth excited (CT) state.
Table S41	Atomic coordinates and partial charges of dimer *a*602-*a*603 in its sixth excited (CT) state.
Table S42	Atomic coordinates and partial charges of dimer *a*603-*a*608 in its ground state.

Worksheet	Data presented
Table S43	Atomic coordinates and partial charges of dimer *a*603-*a*608 in its first excited state.
Table S44	Atomic coordinates and partial charges of dimer *a*603-*a*608 in its sixth excited (CT) state.
Table S45	Atomic coordinates and partial charges of dimer *a*608-*a*614 in its ground state.
Table S46	Atomic coordinates and partial charges of dimer *a*608-*a*614 in its first excited state.
Table S47	Atomic coordinates and partial charges of dimer *a*608-*a*614 in its fifth excited (CT) state.
Table S48	Atomic coordinates and partial charges of dimer *a*608-*a*614 in its sixth excited (CT) state.
Table S49	Atomic coordinates and partial charges of dimer *a*604-*b*605 in its ground state.
Table S50	Atomic coordinates and partial charges of dimer *a*604-*b*605 in its first excited state.
Table S51	Atomic coordinates and partial charges of dimer *a*604-*b*605 in its fifth excited (CT) state.
Table S52	Atomic coordinates and partial charges of dimer *a*604-*b*605 in its sixth excited (CT) state.
Table S53	Atomic coordinates and partial charges of dimer *a*604-*b*605 in its eighth excited (CT) state.
Table S54	Atomic coordinates and partial charges of dimer *a*608-*b*607 in its ground state.
Table S55	Atomic coordinates and partial charges of dimer *a*608-*b*607 in its first excited state.
Table S56	Atomic coordinates and partial charges of dimer *a*608-*b*607 in its fifth excited (CT) state.
Table S57	Atomic coordinates and partial charges of dimer *a*609-*b*607 in its ground state.
Table S58	Atomic coordinates and partial charges of dimer *a*609-*b*607 in its first excited state.
Table S59	Atomic coordinates and partial charges of dimer *a*609-*b*607 in its fifth excited (CT) state.
Table S60	Atomic coordinates and partial charges of dimer *b*607-*b*615 in its ground state.
Table S61	Atomic coordinates and partial charges of dimer *b*607-*b*615 in its first excited state.
Table S62	Atomic coordinates and partial charges of dimer *b*607-*b*615 in its seventh excited (CT) state.
Table S63	Atomic coordinates and partial charges of dimer *a*609-*a*611 in its ground state.
Table S64	Atomic coordinates and partial charges of dimer *a*609-*a*611 in its first excited state.
Table S65	Atomic coordinates and partial charges of dimer *a*609-*a*611 in its fifth excited (CT) state.
Table S66	Atomic coordinates and partial charges of dimer *a*610-*a*611 in its ground state.
Table S67	Atomic coordinates and partial charges of dimer *a*610-*a*611 in its first excited state.
Table S68	Atomic coordinates and partial charges of dimer *a*610-*a*611 in its fifth excited (CT) state.
Table S69	Atomic coordinates and partial charges of dimer *a*610-*a*611 in its sixth excited (CT) state.
Table S70	Atomic coordinates and partial charges of dimer *a*612-*a*613 in its ground state.
Table S71	Atomic coordinates and partial charges of dimer *a*612-*a*613 in its first excited state.
Table S72	Atomic coordinates and partial charges of dimer *a*612-*a*613 in its fifth excited (CT) state.
Table S73	Atomic coordinates and partial charges of dimer *a*612-*a*613 in its sixth excited (CT) state.

Worksheet	Data presented
Table S74	Atomic coordinates and partial charges of dimer *a*612-*a*613 in its seventh excited (CT) state.
Table S75	Atomic coordinates and partial charges of violaxanthin (XAT617) in its ground state.
Table S76	Atomic coordinates and partial charges of lutein (LUT616) in its ground state.
Table S77	Atomic coordinates and partial charges of beta carotene (BCR618) in its ground state.
